# CT Brain Perfusion in the Prediction of Final Infarct Volume: A Prospective Study of Different Software Settings for Acute Ischemic Core Calculation

**DOI:** 10.3390/diagnostics12102290

**Published:** 2022-09-22

**Authors:** Karin Kremenova, Jiri Lukavsky, Michal Holesta, Tomas Peisker, David Lauer, Jiri Weichet, Hana Malikova

**Affiliations:** 1 Radiology Department, Third Faculty of Medicine, Charles University, Faculty Hospital Kralovske Vinohrady, 100 34 Prague, Czech Republic; 2 Institute of Psychology, Czech Academy of Sciences, 110 00 Prague, Czech Republic; 3 Neurology Department, Third Faculty of Medicine, Charles University, Faculty Hospital Kralovske Vinohrady, 100 34 Prague, Czech Republic; 4 Institute of Anatomy, Second Faculty of Medicine, Charles University, 150 00 Prague, Czech Republic

**Keywords:** stroke imaging, magnetic resonance imaging, endovascular thrombectomy, penumbra, syngo.via

## Abstract

CT perfusion (CTP) is used for the evaluation of brain tissue viability in patients with acute ischemic stroke (AIS). We studied the accuracy of three different syngo.via software (SW) settings for acute ischemic core estimation in predicting the final infarct volume (FIV). The ischemic core was defined as follows: Setting A: an area with cerebral blood flow (CBF) < 30% compared to the contralateral healthy hemisphere. Setting B: CBF < 20% compared to contralateral hemisphere. Setting C: area of cerebral blood volume (CBV) < 1.2 mL/100 mL. We studied 47 AIS patients (aged 68 ± 11.2 years) with large vessel occlusion in the anterior circulation, treated in the early time window (up to 6 h), who underwent technically successful endovascular thrombectomy (EVT). FIV was measured on MRI performed 24 ± 2 h after EVT. In general, all three settings correlated with each other; however, the absolute agreement between acute ischemic core volume on CTP and FIV on MRI was poor; intraclass correlation for all three settings was between 0.64 and 0.69, root mean square error of the individual observations was between 58.9 and 66.0. Our results suggest that using CTP syngo.via SW for prediction of FIV in AIS patients in the early time window is not appropriate.

## 1. Introduction

Endovascular thrombectomy (EVT) provides the best clinical outcome in patients with acute ischemic stroke (AIS) due to large vessel occlusion (LVO) in the anterior circulation, in both early (within 6 h) and late (beyond 6 h) periods from onset [1,2,3,4]. In addition to non-enhanced CT (NECT) and CT angiography (CTA), CT perfusion (CTP) has become an inseparable part of ischemic “code-stroke” protocols in some centers. It is believed that CTP is able to distinguish non-salvageable, irreversible ischemic brain tissue (core) from salvageable, reversible ischemia (penumbra) [5,6]. Moreover, CTP results are included in the EVT guidelines in patients with AIS with LVO in the anterior circulation presenting beyond the 6 h time window from onset and in patients with unknown time of last known well (LKW) [1,3].

Over the years, several post-processing software (SW) packages for CTP have been developed by different vendors, e.g., RAPID (iSchemaView Inc., Menlo Park, CA, USA), syngo.via CT Neuro Perfusion (Siemens Healthineers, Erlangen, Germany), Olea (Olea Medical, La Ciotat, France), and IntelliSpace Portal CT Brain Perfusion (Royal Philips Healthcare, Best, The Netherlands). RAPID is used by more than 1000 institutions worldwide, and studies using RAPID SW were decisive for the adjustment of the guidelines for EVT in the late time window [3,7]. However, not all hospitals have access to RAPID and use SW from various vendors. According to a number of recent studies, the use of various SW platforms may be problematic due to the significant discrepancy in estimates of the ischemic core and penumbra [8,9,10,11,12,13,14]. In some cases, CTP results are used as a relevant criterion for treatment decision making; SW-generated volumes should be consistent regardless of the platform. There is an effort to adjust the calculation parameters to achieve the most accurate results with regard to the final infarct volume (FIV).

We have been studying diagnostic and therapeutic possibilities in AIS patients for several years [15,16,17]. In the present study, we evaluated the accuracy of three different syngo.via settings in the estimation of the acute ischemic core on CTP. We compared the CTP ischemic core volume calculated according to different settings with FIV measured on magnetic resonance imaging (MRI) performed 24 ± 2 h after successful EVT. We hypothesized that the FIV measured on MRI would be similar or slightly larger (due to ischemic growth) than the estimated ischemic core volume on CTP. 

## 2. Materials and Methods

### 2.1. Study Design and Patient Selection

Our prospective, single-center study was initiated in January 2020 and terminated in May 2022. The study was approved by the Institutional Ethics Committee. Signed, informed consent was provided by all patients to participate in the study. 

We enrolled consecutive patients who suffered from AIS with LVO in the anterior circulation (occlusion of the M1 or M2 segments of the medial cerebral artery or occlusion of the internal carotid artery) indicated for EVT according to neurological guidelines [1]. Further inclusion criteria were as follows:

- Underwent technically successful EVT immediately after initial CT;
- Technically compliant initial CTP study available;
- Underwent MRI 24 ± 2 h after EVT;
- Agreed with enrolment in the study and provided signed, informed consent.

Exclusion criteria were as follows:

- Unsuccessful EVT: TICI (Thrombolysis in cerebral infarction scale) < 2b; periprocedural complications such as arterial dissection, intracranial hemorrhage, etc.;
- Inability to undergo MRI follow-up due to poor overall condition of a patient after EVT, contraindications to MRI as pacemakers, implantable cardioverter-defibrillator, or claustrophobia;
- Patient disagreement with enrolment in the study or MRI follow-up.

In all patients, the National Institute of Health Stroke Scale (NIHSS) for assessing the severity of AIS was evaluated by a neurologist, and all time data in relation to AIS and its management were recorded [18]. 

### 2.2. Initial CT Imaging and Post-Processing

All patients were imaged using a multidetector dual source Somatom Drive scanner (Siemens Healthineers, Erlangen, Germany). All subjects initially underwent our institutional standard imaging stroke protocol consisting of NECT, CTA, and CTP. On NECT, acute ischemic changes were evaluated using the Alberta stroke program early CT score (ASPECTS) by 2 neuroradiologists (with 25 and 5 years’ experience) [19]. On CTA, the presence of occlusion and laterality were assessed. CTA covering precerebral and cerebral arteries (from aortic arch to vertex) was performed after the administration of a contrast agent with a concentration of 400 mg/mL intravenously by a power injector with the following parameters: 50 mL with a flow of 3 mL/s followed by saline flush. CTP covering 100 mm in the *z*-axis (the entire supratentorial anatomy of the brain) was performed after the administration of a contrast agent with a concentration of 400 mg/mL intravenously by a power injector with the following parameters: 50 mL with a flow of 5 mL/s followed by saline flush. CTP was assessed out of the acute setting and EVT was indicated regardless of CTP results. Detailed technical parameters of CT scans are provided in Table 1.

CTP post-processing was performed on a dedicated syngo.via workstation (NeuroPerfusion suite, version VB30A). We used the following three different settings to calculate the volume of the ischemic core and penumbra:

- Setting A (our adjustment of default syngo.via settings)

The ischemic core was defined as an area with cerebral blood flow (CBF) < 30% in comparison with the contralateral hemisphere without acute ischemia. The penumbra was defined as an area with a time to maximum (TMAX) > 6 s. TMAX was defined as the time from inflow of an intraarterial contrast agent to the area of interest to the peak density in the area.

- Setting B (adjustment of syngo.via settings by Siemens professionals)

The ischemic core was defined as an area with CBF < 20% in comparison with the contralateral hemisphere without acute ischemia. The penumbra was defined as an area with TMAX > 6 s.

- Setting C (default syngo.via settings)

The ischemic core was defined as an area with cerebral blood volume (CBV) < 1.2 mL/100 mL. The penumbra was defined as an area with CBF < 27 mL/100 mL/min.

### 2.3. Follow-Up MRI and Evaluation

Follow-up MRI was performed 24 ± 2 h after successful EVT. MRI scans were performed on a 1.5T MRI scanner (Signa HDx 1.5 T, GE Healthcare, Milwaukee, WI, USA) using an HD 8 Channel High-Resolution Brain Array Coil. The imaging protocol consisted of the following axial sequences: T2 fluid-attenuated inversion recovery (FLAIR), T2 gradient echo (GRE), diffusion-weighted imaging (DWI) (b-value 0 and 1000, ADC map), 3D time of flight angiography (TOF). No contrast agent was applied. The technical parameters of all MRI sequences are provided in Table 2.

A visible hyperintense area on both T2 FLAIR and DWI images with ADC decrease was assessed as the final infarct. Manual segmentation of the final infarct was performed on syngo.via volumetric measurement SW using 5 mm slices. All manual segmentations and measurements were performed by one neuroradiologist, who was blinded to the CTP results. 

Recanalization stability was confirmed using a 3D TOF sequence with MIP 3D reconstruction. 

### 2.4. Statistical Analysis 

In our analyses, we first compared the ischemic core volumes obtained via Settings A, B, and C using Pearson correlation. Our primary question was whether the values of ischemic core volumes (for any syngo.via setting) numerically correspond to the FIV measured on follow-up MRI. To measure this agreement, we used intraclass correlation (ICC) and its 95% confidence interval (two-way mixed effects model for absolute agreement, single measurement) and root mean square error of the individual observations (RMSE). We include Bland–Altman plots to visualize the agreement. To describe how well CTP values predicted the FIV (without the requirement of absolute agreement), we report Pearson correlation coefficients (high correlation is necessary but not a sufficient requirement for high agreement). Additionally, we also compared the penumbra volumes from each setting with Pearson correlation. We used the threshold α = 0.05 for statistical significance.

## 3. Results

### 3.1. Patient Selection

During the study period, 82 patients with AIS due to LVO in the anterior circulation who were indicated to EVT underwent a complete imaging protocol including CTP. However, 35 patients were excluded for the following reasons: in 10 patients EVT was technically unsuccessful or with periprocedural complications; 18 patients were not able to undergo MRI due to their poor overall condition; 2 patients had contraindications to MRI; 3 patients refused to undergo MRI; and in 2 patients, the CTP scan was of low quality and core calculation was not possible. 

A total of 47 patients (15 females, 32 males) were included in the study. Table 3 summarizes relevant patient clinical and demographic data.

### 3.2. CTP and MRI Data 

CTP data:

- Setting A (CBF < 30%, TMAX > 6 s): The median ischemic core volume was 34 mL (IQR 46.5 mL).
- Setting B (CBF < 20%, TMAX > 6 s): The median ischemic core volume was 10 mL (IQR 24 mL).
- Setting C (CBV < 1.2 mL/100 mL, CBF < 27 mL/100 mL/min): The median ischemic core volume was 23 mL (IQR 22 mL).

MRI data: 

The median FIV was 20 mL (IQR 44.1 mL). Recanalization stability was confirmed in 46 patients (97.9%). 

### 3.3. Agreement of CTP and MRI Data

**Figure 1:**
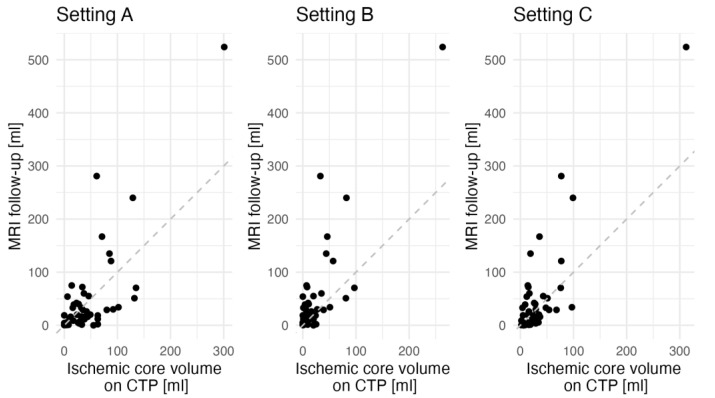
Comparison of ischemic core volume on CT perfusion with final infarct volume on magnetic resonance imaging follow-up for three different settings.

In general, all three settings correlated each other (see Figure 1; Settings A–B: r = 0.963; A–C: r = 0.886, B–C: r = 0.925; all *p*’s < 0.001). 

**Figure 2:**
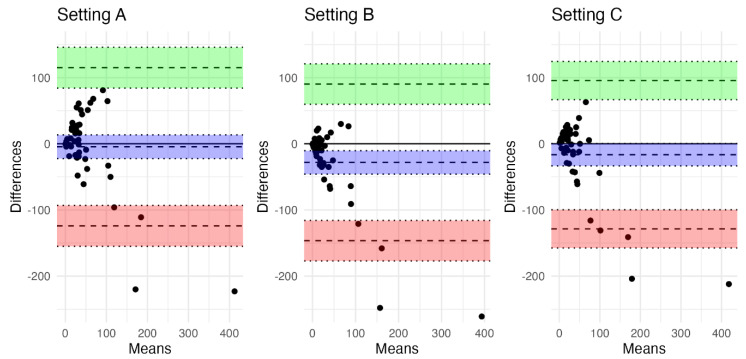
Bland–Altman plots comparing ischemic core volume on CT perfusion for each setting and the final infarct volume on magnetic resonance imaging follow-up. Shaded areas represent 95% confidence intervals for the upper/lower limit of agreement (green/red) and for bias (blue).

**Figure 3:**
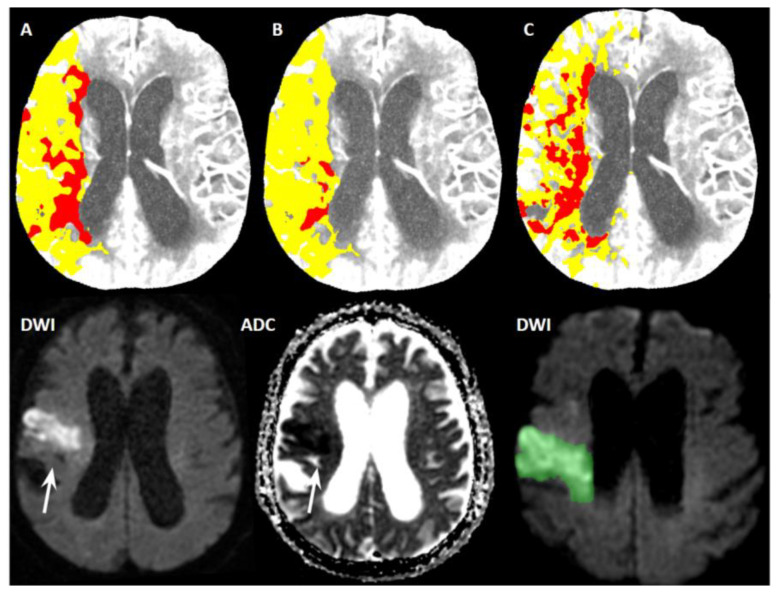
Comparison of three different syngo.via software settings with final infarct volume on magnetic resonance imaging follow-up in an exemplary patient. A 75-year-old man with acute ischemic stroke due to right middle cerebral artery occlusion confirmed on CT angiography. CT perfusion maps (upper row) obtained using different syngo.via software settings depicting the different extent of ischemic core (red area) and penumbra (yellow area). Using Setting A, the ischemic core was 35 mL (**A**), for Setting B, the ischemic core was 6 mL (**B**) and for Setting C, the ischemic core was 54 mL (**C**). Magnetic resonance imaging follow-up (lower row) 24 h after endovascular treatment depicted the final infarct as a hyperintense area on diffusion-weighted images (DWI) with a hypointense area on an apparent diffusion coefficient (ADC) map (arrow). Final infarct volume (green area) measured on magnetic resonance imaging was 29 mL.

However, they were not very tightly related to FIV measured on follow-up MRI. Setting B had the weakest agreement (ICC = 0.642, 95% confidence interval CI [0.438, 0.784], RMSE = 66.0), but the differences between Setting A (ICC = 0.668, 95% CI [0.473, 0.8], RMSE = 60.5) and Setting C (ICC = 0.693, 95% CI [0.509, 0.817], RMSE = 58.9) were negligible. The corresponding Bland–Altman plots are depicted in Figure 2. See also Figure 3 for a better understanding. 

When inspecting the correlations (i.e., dropping the requirement of absolute agreement), we found the highest correlations in Settings B and C (B: r = 0.841, C: r = 0.846) relative to Setting A (r = 0.775; all *p*-values < 0.001). Altogether, the correlation between individual settings and follow-up MRI was high, but hardly indicative of good absolute agreement. Given our sample size, the differences were too small to select which settings corresponded best with MRI values. Although we found that settings were highly correlated for ischemic core volumes, the correlations were much lower for penumbra volumes (Settings A–B: r = 0.767, *p* < 0.001; A–C: r = 0.288, *p* = 0.049; B–C: r = 0.326; *p* = 0.025). 

Our dataset included six patients with MRI volumes above 120 mL. As more extreme values could affect the results (inflate RMSE or increase correlation), we reran the analysis on a smaller dataset excluding these six measurements. As expected, we observed smaller RMSE errors, but more importantly both the agreement (ICC values) and correlations with MRI values dropped (Setting A: ICC = 0.322, *p* = 0.019; RMSE = 34.5; r = 0.351, *p* = 0.024; Setting B: ICC = 0.449, *p* = 0.001; RMSE = 23.8; r = 0.45, *p* = 0.003; Setting C: ICC = 0.331, *p* = 0.016; RMSE = 24.3; r = 0.323, *p* = 0.04). To summarize, even on smaller volumes below 120 mL, we saw relatively large mean errors over 20 and both lower agreement and correlation with MRI measurements.

When visually checking for bias, we concluded that Setting A as well as Setting C showed an overestimation of FIV in absolute values in 25 patients (53.2%), whereas Setting B showed an overestimation in 12 patients (25.5%). 

## 4. Discussion

The purpose of our study was to compare three different syngo.via CTP settings for ischemic core volume calculation and to evaluate their accuracy in the prediction of FIV. Our study group consisted of AIS patients with LVO in the anterior circulation treated with technically successful EVT, and FIV was assessed on MRI 24 h after EVT. We were interested in absolute agreement between the ischemic core volume on CTP and FIV on MRI. We hypothesized that the ischemic core volume on CTP would be the same or slightly smaller than FIV. According to our results, all three settings correlated with each other and the correlation between individual settings and MRI follow-up was high; however, this was hardly indicative of good absolute agreement. The absolute agreement between acute ischemic core on CTP and FIV on MRI was poor, even after the exclusion of extreme values from the calculation. ICC for all three settings was between 0.64 and 0.69 and RMSE was between 58.9 and 66.0. After the exclusion of extreme values, ICC improved to 0.32–0.45 and RMSE to 23.9–34.5; however, this was still unsatisfactory. According to statistical analysis, the best agreement between the ischemic core volume on CTP and FIV was achieved using Setting C (the default syngo.via setting). The weakest agreement was achieved using Setting B (syngo.via adjustment by Siemens professionals)., This setting overestimated the FIV in 25.5% of patients in contrast to Settings A and C with an overestimation of FIV in about 53% of patients. We hypothesized that FIV would not be less than the estimated ischemic core volume on CTP, as we assume that the ischemic core is non-salvageable brain tissue. 

Overestimation of the FIV on initial CTP, especially in patients with a short time window from LKW to CTP, has been proven in some recent studies [15,20,21,22,23]. The term ghost infarct core (GIC) was established for this phenomenon and it is considered to be one of the greatest limitations of CTP, which could lead to the erroneous exclusion of some patients from causative treatment, which is in the case of LVO EVT [18,20,21,24]. Moreover, advanced methods such as CTP are also used in selecting patients for intravenous thrombolysis in a time window longer than 4.5 h or in wake-up strokes [25]. Again, there is a risk that a substantial number of patients could be excluded from treatment. However, our study does not support the presence of GIC; although, our patients were imaged in the early time window and had fast reperfusion; mean time from LKW to EVT was only 213 ± 86 min, and mean time between LKW and CT was 126 ± 83 min. It must be noted that the above-mentioned studies reported GIC had important limitations, as they used only NECT follow-up for the evaluation of FIV. That approach was likely error-prone as MRI has been shown to be the most accurate diagnostic method for assessing acute ischemic changes [26,27,28,29]. Due to consistent MRI follow-up, we consider our volumetric FIV data to be more reliable; moreover, all subjects underwent MRI within the same time interval, scans were performed on the same 1.5T MRI scanner and the FIV was measured by one neuroradiologist.

Several recent comparative studies have shown differences in the ischemic core and penumbra volumes on CTP depending on the postprocessing SW [7,9,30,31]. The discrepancy in volumes between various SW is due to differences in acquisition techniques, contrast agent delivery, thresholds for calculation of the ischemic core and penumbra, reference values for grey and white matter, differences in post-processing steps such as smoothing, and defining arterial input and venous output functions [8,9]. RAPID SW, which was used in many studies including the multicenter DEFUSE 3 study, is considered the most reliable [3,30,32]. In a study by Austein et al. focusing on the accuracy of commercial CTP SW in predicting FIV, RAPID showed the highest accuracy in the prediction of FIV in comparison to other SW used in clinical practice [30]. Koopman et al. conducted a study to compare the results of different syngo.via settings with RAPID SW results [9]. When they used the default syngo.via setting (Setting C in the present study) with the ischemic core defined as CBV < 1.2 mL/100 mL, ischemic core volumes were larger in comparison with RAPID. They then used the same setting as in RAPID (Setting A in the present study) with the ischemic core defined as CBF < 30% in comparison with the contralateral hemisphere without acute ischemia; however, core volumes were significantly larger in comparison with the RAPID calculation. However, their study had some substantial limitations. For the measurement of FIV, they used a 5–7-day NECT follow-up. It is well known that CT sensitivity for acute ischemic changes is lower than MRI sensitivity [26,27,28,29]. Moreover, the follow-up was conducted 5–7 days after EVT, which is an important difference from our follow-up, which was conducted one day after EVT. According to our opinion, the measurement of FIV in their study may have been affected by vasogenic edema. Xiong et al. compared Olea and RAPID SW. In their study, RAPID showed a greater correlation of ischemic core volume with FIV on MRI than Olea in patients with technically successful EVT [7].

Although our study had a prospective design, there are some important limitations. The sample of studied subjects was relatively small and our research was conducted as a single center study. The vast majority of subjects were treated in the early time window; therefore, we do not have subjects treated in the extended time window for comparison. Moreover, RAPID SW was not available for comparison. 

## 5. Conclusions

In the present prospective study, the absolute agreement between acute ischemic core volume on CTP and FIV on MRI follow-up after technically successful EVT was poor. Despite the use of three different syngo.via SW settings, none were accurate enough in the satisfying prediction of the FIV. Our results suggest that using CTP syngo.via SW to predict FIV in AIS patients imaged and treated in the early time window is not appropriate, as there is a non-negligible risk of excluding a substantial number of patients from causative treatment.

## Figures and Tables

**Table 1:** Acquisition and reconstruction parameters of non-enhanced CT, CT angiography, and CT perfusion.

Parameter	NECT	CTA	CTP
Rotation time (s)	1	0.28	0.28
Tube voltage (kVp)	120	120 **	70
Tube current (mAs)	273 *	84 *	200
Gantry tilt	no	no	no
Field of view (mm)	250	250	200
Iterative reconstruction	Yes	Yes	No
Reconstruction filter	Soft tissue	Soft tissue	Soft tissue
Primary reconstruction slice thickness/increment (mm)	3/3	0.75/0.5	1.5/1
Multiplanar reconstruction slice thickness/increment (mm)	3/3	10/2	-

* Scan performed with tube current modulation, reference value given; ** Scan performed with tube voltage modulation, reference value given. CTA, CT angiography; CTP, CT perfusion; NECT, non-enhanced CT.

**Table 2:** Acquisition parameters of magnetic resonance imaging.

Sequence	TR (ms)	TE (ms)	TI (ms)	Slice Thickness (mm)
**T2 FLAIR**	8500	123.66	2000	5
GRE T2 *	800	20	-	5
**DWI**	8000	20	-	5
**3D TOF**	27	6.8	-	1.2

DWI, diffusion-weighted imaging; FLAIR, fluid-attenuated inversion recovery; GRE, gradient echo; TE, echo time; TI, inversion time; TOF, time of flight angiography; TR, repetition time.

**Table 3:** Relevant initial clinical, imaging, and stroke treatment data.

		No. of Patients (%)	Mean ± SD
Age		-	68 ± 11.2
Sex	Female	15 (32)	-
	Male	32 (68)	-
Occluded artery	M1 segment of MCA	21 (45)	-
	M2 segment of MCA	12 (26)	-
	ICA	3 (6)	-
	Tandem occlusion	11 (23)	-
Side of occlusion	Right	21 (45)	-
	Left	26 (55)	-
ASPECTS		-	7.5 ± 1.9
NIHSS		-	13.5 ± 5.1
LKW-CT time (min)		-	126 ± 83
CT-groin puncture time (min)		-	50 ± 16
LKW-recanalization time (min)		-	213 ± 86
Bridging IVT		29 (62)	

ASPECTS, the Alberta stroke program early CT score; EVT, endovascular thrombectomy; ICA, internal carotid artery; IVT, intravenous thrombolysis; LKW, last known well; MCA, medial cerebral artery; NIHSS, the National Institute of Health Stroke Scale; No, number; SD, standard deviation.

## Data Availability

Data are available upon a reasonable request to the study investigators.
